# Identification of immune-related LncRNA for predicting prognosis and immunotherapeutic response in bladder cancer

**DOI:** 10.18632/aging.104115

**Published:** 2020-11-18

**Authors:** Yucai Wu, Lei Zhang, Shiming He, Bao Guan, Anbang He, Kunlin Yang, Yanqing Gong, Xuesong Li, Liqun Zhou

**Affiliations:** 1 Department of Urology, Peking University First Hospital, Beijing, China; 2 Institute of Urology, Peking University, Beijing, China; 3 National Urological Cancer Center, Beijing, China; 4 Urogenital Diseases (Male) Molecular Diagnosis and Treatment Center, Peking University, Beijing, China

**Keywords:** lncRNAs, bladder cancer, survival, immune infiltration, immunotherapy

## Abstract

Long noncoding RNAs (lncRNAs) have multiple functions in the cancer immunity response and the tumor microenvironment. To investigate the immune-related lncRNA (IRlncRNA) signature for predicting prognosis and immunotherapeutic response in bladder cancer (BLCA), we extracted BLCA data from The Cancer Genome Atlas (TCGA) database. Finally, a total of 405 cases were enrolled and 8 prognostic IRlncRNAs (*MIR181A2HG, AC114730.3*, *LINC00892*, *PTPRD-AS1, LINC01013, MRPL23-AS1,* *LINC01395*, *AC002454.1)* were identified in the training set. Risk scores were calculated to divide patients into high-risk and low-risk groups, and the high-risk patients tended to have a poor overall survival (OS). Multivariate Cox regression analysis confirmed that the IRlncRNA signature could be an independent prognostic factor. The results were subsequently confirmed in the validating set. Additionally, this 8-IRlncRNA classifier was related to recurrence free survival (RFS) of BLCA. Functional characterization revealed this signature mediated immune-related phenotype. This signature was also associated with immune cell infiltration (i.e., macrophages M0, M2, Tregs, CD8 T cells, and neutrophils) and immune checkpoint inhibitors (ICIs) immunotherapy-related biomarkers [mismatch repair (MMR) genes, tumor mutation burden (TMB) and immune checkpoint genes]. The present study highlighted the value of the 8-IRlncRNA signature as a predictor of prognosis and immunotherapeutic response in BLCA.

## INTRODUCTION

Bladder cancer (BLCA) is the fourth most prevalent cancer in men and the most frequently diagnosed malignancy of the urinary system worldwide [1, 2]. Nonmetastatic bladder cancer is separated into non-muscle-invasive bladder cancer (NMIBC) and muscle-invasive bladder cancer (MIBC). However, approximately 25% of BLCA patients are diagnosed with MIBC or metastatic disease [3, 4]. In addition, BLCA has a high recurrence rate, and approximately half of patients relapse after radical surgery and present with metastases [5, 6]. Platinum-based chemotherapy and new ICIs has provided unprecedented benefits for patients with metastatic urothelial carcinoma, but the heterogeneous properties of BLCA contribute to different clinical outcomes for BLCA patients with current standard therapy [7]. To improve survival and reduce the burden of BLCA, researchers must develop novel biomarkers for better prediction of the prognosis and treatment response of BLCA.

Long noncoding RNAs (lncRNAs) are a class of non-coding transcripts more than 200 nucleotides in length [8]. It has been suggested that lncRNAs function as key players in post-transcriptional regulatory mechanisms that target mRNA splicing, stability, or translation [9]. Alterations in lncRNA expression and mutations are closely associated with tumorigenesis, tumor progression and metastasis, highlighting the emerging roles of lncRNAs as novel biomarkers and therapeutic targets for cancer [10, 11]. Increasing evidence also suggests that LncRNAs play fundamental roles in regulating genes encoding products involved in cancer immunity [12]. For instance, NKILA lncRNA promotes tumor immune evasion by sensitizing T cells to activation-induced cell death [13]. Lnc-chop promoted the immunosuppressive function of myeloid-derived suppressor cells in tumor environment by activating C/EBPβ and upregulating the expression of arginase-1, NO synthase 2, NADPH oxidase 2, and cyclooxygenase-2 [14]. Using CRISPR-Cas9 to target lncRNA UCA1 and in turn block PD-1 function can enhance antitumor activity in BLCA patients [15]. Although immune-related lncRNAs have been identified as potential biomarkers, research involving immune-related lncRNA signatures in survival and treatment of BLCA is lacking [16, 17].

The initial assessment of BLCA has been explored recently. In clinical practice, lncRNAs, miRNAs and clinicopathological factors including TNM stage and lymph node status have been gradually used to assess the prognosis of BLCA. Recent research has revealed that the IRlncRNA signature is associated with the prognosis and immunotherapy of BLCA patients [18]. Therefore, we attempted to identify a number of IRlncRNAs as potential biomarkers to predict the outcome of BLCA. We constructed an 8-IRlncRNA classifier for OS by using the least absolute shrinkage and selection operator (LASSO) method and multivariable Cox regression. In addition, this 8-IRlncRNA classifier was strongly related to RFS in BLCA. Furthermore, our classifier was associated with immune cell infiltration and the response to ICIs treatment. Our results demonstrated that the 8-IRlncRNA classifier could serve as a reliable prognostic predictor of BLCA survival and ICIs immunotherapy.

## RESULTS

### Data source and processing

**Figure 1:**
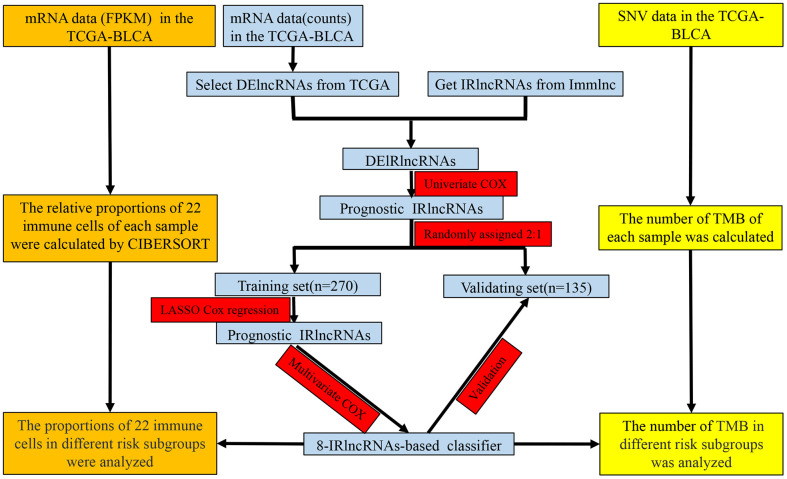
**Study flowchart showing the process of constructing the 8-IRlncRNA classifier to predict prognosis of BLCA.** BLCA, bladder cancer; FPKM, Fragments per Kilobase Million; SNV, single nucleotide variation; TMB, tumor mutation burden; DElncRNAs, differentially expressed lncRNAs; IRlncRNA, immune-related LncRNA.

**Figure 2:**
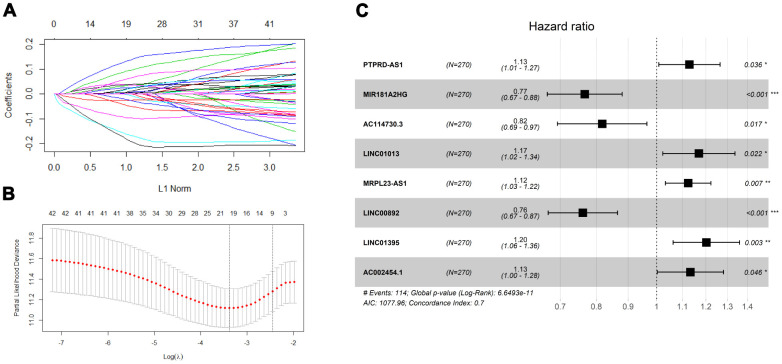
**Screening prognosis immune-related lncRNA for model construction.** (**A**) Validation was performed for tuning parameter selection through the least absolute shrinkage and selection operator (LASSO) regression model for overall survival (OS). (**B**) Elucidation for LASSO coefficient profiles of prognostic lncRNAs. (**C**) Forest plot exhibited the hazard ratio (HR) with 95% confidence interval (95% CI) of prognostic immune-related lncRNA in BLCA on the basis of the multivariate Cox regression result.

As shown in Figure 1, the overlap was taken from 2420 IRlncRNAs and 1648 differentially expressed lncRNAs (DElncRNAs), and 190 differentially expressed IRlncRNAs (DEIRlncRNAs) were retained. Then, univariate Cox regression was conducted to choose characteristics for prognostic prediction of patients, and 42 DEIRlncRNAs with p<0.05 were retained for further analysis. The clinical characters of BLCA patients were downloaded from the UCSC database, and subsequently, TCGA dataset was split randomly into a training set (n=270) and a validating set (n=135) at a 2:1 ratio. There were no significant differences in age, gender, histologic grade, pathological stage, or diagnosis subtype between the two groups (Table 1). Then we identified 8 IRlncRNAs that were strongly associated with OS of BLCA by LASSO (Figure 2A, 2B) and multivariate Cox regression analysis in the training set. The forest plot shows the hazard radio (HR) with 95% confidence interval (CI) of eight IRlncRNAs (Figure 2C) and detailed information on these lncRNAs is listed in Table 2. Higher expression of *MIR181A2HG* (HR: 0.77 [95%CI 0.67-0.88], p<0.001)*, AC114730.3* (HR: 0.82 [95%CI 0.69-0.97], p=0.017) and *LINC00892* (HR: 0.76 [95%CI 0.67-0.87], p<0.001) tended to predict increased survival, while higher expression of *PTPRD-AS1* (HR: 1.13 [95%CI 1.01-1.27], p=0.036)*, LINC01013* (HR: 1.17 [95%CI 1.02-1.34], p=0.022)*, MRPL23-AS1* (HR: 1.12 [95%CI 1.03-1.22], p=0.007)*,* *LINC01395* (HR: 1.20 [95%CI 1.06-1.36], p=0.003) and *AC002454.1* (HR: 1.13 [95%CI 1.00-1.28], p=0.046) tended to predict decreased survival.

**Table 1:** Clinical features of BLCA patients in the training and validating sets.

**Features**	**Training set (n=270)**	**Validating set (n=135)**	**Pearson x2**	**P**
Age (years), no (%)				
≤70	147(54.4)	82(60.7)		
>70	123(45.6)	53(39.3)	1.452	0.228
Gender, no (%)				
Male	202(74.8)	98(72.6)		
Female	68(25.2)	37(27.4)	0.231	0.630
Pathological stage, no (%)				
I+II	95(35.2)	38(28.1)		
III+IV	175(64.8)	97(71.9)	2.021	0.155
Histologic grade, no (%)				
NA	1(0.4)	2(1.5)		
Low	13(4.8)	8(5.9)		
High	256(94.8)	125(92.6)	1.643	0.440
Diagnosis subtype, no (%)				
NA	3(1.1)	1(0.7)		
Non-Papillary	179(66.3)	91(67.4)		
Papillary	88(32.6)	43(31.9)	0.163	0.922

Abbreviations: BLCA, Bladder cancer; NA, Not available.

**Table 2:** Eight immune-related lncRNAs significantly associated with the OS of BLCA patients in the training set.

**Gene symbol**	**Description**	**Coefficient**	Immune pathway*
PTPRD-AS1	PTPRD Antisense RNA 1	0.121637	Cytokines
MIR181A2HG	MIR181A2 Host Gene	-0.26599	Cytokines
AC114730.3	No data available	-0.20142	Antigen Processing and Presentation
LINC01013	Long Intergenic Non-Protein Coding RNA 1013	0.156705	Cytokines
MRPL23-AS1	MRPL23 Antisense RNA 1	0.117063	TGF-β Family Member
LINC00892	Long Intergenic Non-Protein Coding RNA 892	-0.27288	Antigen Processing and Presentation
LINC01395	Long Intergenic Non-Protein Coding RNA 1395	0.184383	Antimicrobials
AC002454.1	No data available	0.125301	Antigen Processing and Presentation

Abbreviations: BLCA, Bladder cancer; OS, Overall survival.

* Immune pathway was annotated by website Immlnc (http://biobigdata.hrbmu.edu.cn-/ImmLnc/index.jsp).

### An 8-IRlncRNA classifier to predict OS in BLCA

**Figure 3:**
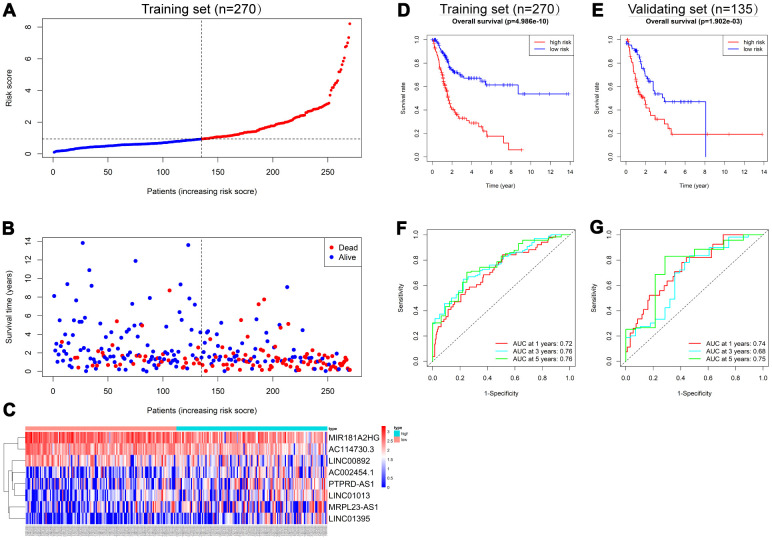
**Construction of the 8-IRlncRNA classifier for predicting prognosis of BLCA.** (**A**) Patients with BLCA were sorted by increasing risk score in the training set. (**B**) Living status of BLCA patients in the training set. (**C**) Heatmap of eight IRlncRNAs expression profiles of different risk groups in the training set. (**D**, **E**) Kaplan-Meier analysis for overall survival (OS) of BLCA patients based on the risk stratification in the training set (**D**) and validating set (**E**). (**F**, **G**) Receiver operating characteristic (ROC) analysis for OS prediction including 1-, 3-, 5-year of BLCA patients in the training set (**F**) and validating set (**G**).

To assess the ability of the IRlncRNA signature to predict the survival of BLCA, we calculated the risk score for each case according to the expression of eight IRlncRNAs: Risk score = (-0.27 * expression value of *MIR181A2HG*) + (-0.20 * expression value of *AC114730.3*) + (-0.27 * expression value of *LINC00892*) + (0.12 * expression value of *PTPRD-AS1*) + (0.16 * expression value of *LINC01013*) + (0.12 * expression value of *MRPL23-AS1*) + (0.18 * expression value of *LINC01395*) + (0.13 * expression value of *AC002454.1*). Cases were split into high-risk and low-risk groups according to the median risk score (Figure 3A) and the mortality was higher in the high-risk group than in the low-risk group in the training set (Figure 3B). Moreover, *MIR181A2HG, AC114730.3* and *LINC00892* were highly expressed in the low-risk group, while *PTPRD-AS1, LINC01013, MRPL23-AS1, LINC01395* and *AC002454.1* were highly expressed in the high-risk group (Figure 3C). The results in the validating set were consistent with the findings described above (Supplementary Figure 1). Survival analysis revealed that patients in the high-risk group had a shorter OS than those in the low-risk group (p<0.001) in the training set (Figure 3D) and a similar result was observed in the validating set (p=0.002) (Figure 3E). Time-dependent receiver operating characteristic (ROC) curves were plotted and the area under curve (AUC) values of the classifier to predict 1-, 3- and 5-year overall survival were 0.72, 0.76, and 0.76, respectively (Figure 3F) in the training set and 0.74, 0.68, and 0.75, respectively, in the validating set (Figure 3G).

### Survival prediction by the 8-IRlncRNA classifier is independent of clinical features

**Figure 4:**
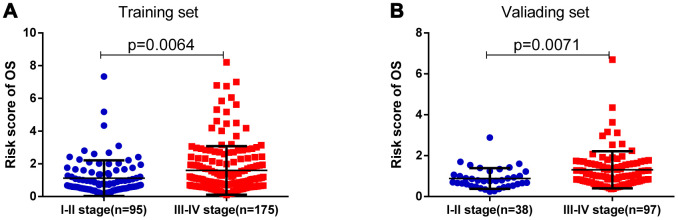
**The risk score was associated with the pathologic stage of BLCA.** (**A**) Boxplot of risk score in patients with different stage in the training set. (**B**) Boxplot of risk score in patients with different stage in the validating set.

As shown in Table 3, clinicopathologic characteristics including age (p=0.038), and pathological stage (p=0.030) showed significant differences between the high-risk group and the low-risk group in the training set, while pathological stage (p=0.009) and diagnosis subtype (p=0.003) displayed distinct differences in the validating set. Patients with advanced pathological stage tended to obtain a high-risk score in the training (p=0.006) and validating groups (p=0.007) (Figure 4).

**Table 3:** Clinicopathological characteristics of the 8-IRlncRNA classifier with OS in the training set and validating set.

**Features**	**Training set(n=270)**				**Validating set(n=135)**		
	**Low risk**	**High risk**	**P**		**Low risk**	**High risk**	**P**
Age (years), no (%)							
≤70	82(60.7)	65(48.1)			43(63.2)	39(58.2)	
>70	53(39.3)	70(51.9)	**0.038**		25(36.8)	28(41.8)	0.550
Gender, no (%)							
Male	34(25.2)	34(25.2)			22(32.4)	15(22.4)	
Female	101(74.8)	101(74.8)	1.000		46(67.6)	52(77.6)	0.194
Pathological stage, no (%)							
I+II	56(41.5)	39(28.9)			26(38.2)	12(17.9)	
III+IV	79(58.5)	96(71.1)	**0.030**		42(61.8)	55(82.1)	**0.009**
Histologic grade no (%)							
NA	1(0.7)	0(0)			1(1.5)	1(1.5)	
Low	10(7.4)	3(2.2)			6(8.8)	2(3.0)	
High	124(91.9)	132(97.8)	0.060		61(89.7)	64(95.5)	0.340
Diagnosis subtype no (%)							
NA	0(0)	3(2.2)			1(1.5)	0(0)	
Non-Papillary	88(65.2)	91(67.4)			37(54.4)	54(80.6)	
Papillary	47(34.8)	41(30.4)	0.099		30(44.1)	13(19.4)	**0.003**

Abbreviations: OS, Overall survival; NA, Not available.

**Figure 5:**
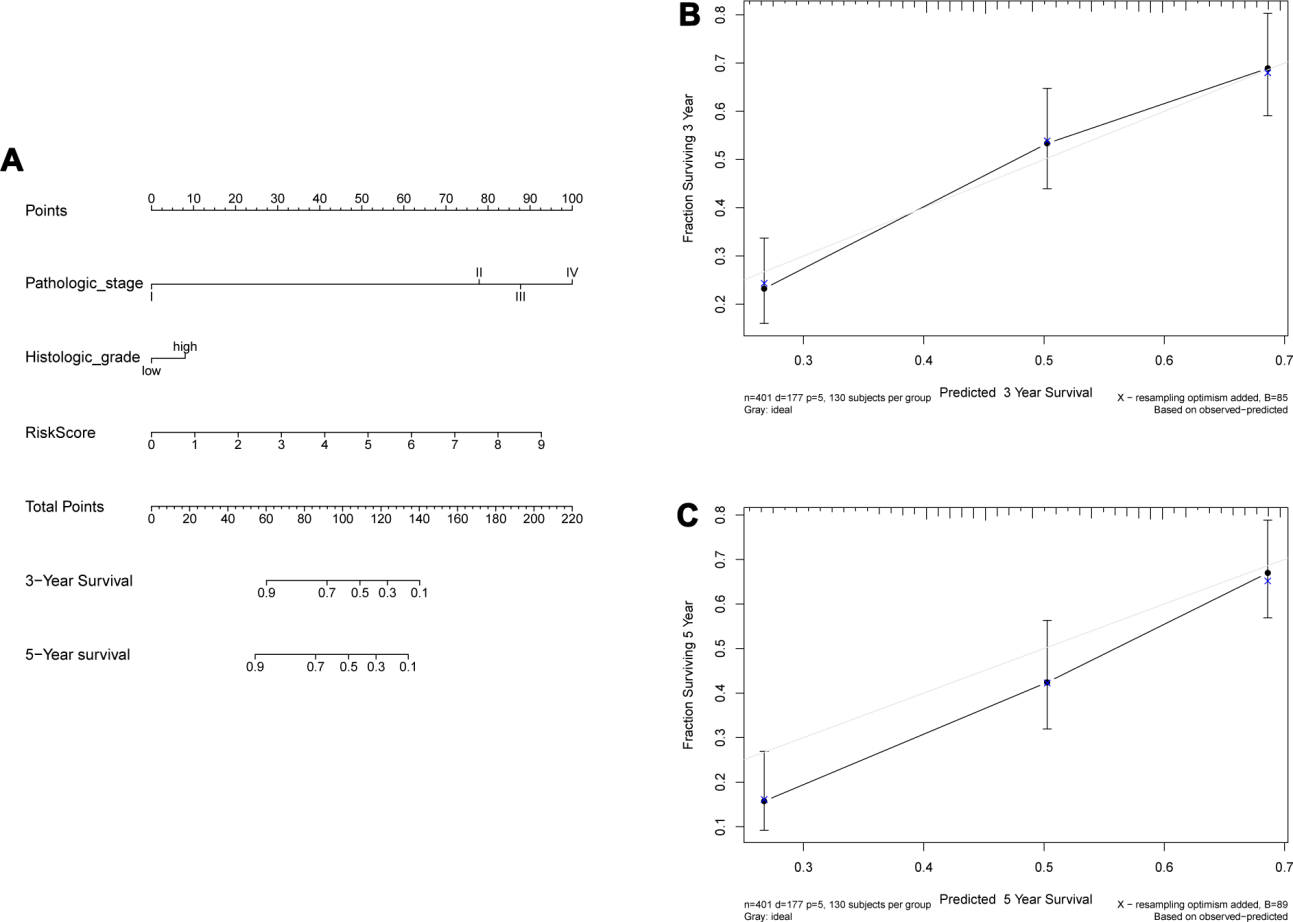
**Construction of a nomogram combined risk score and clinical indicators for predicting survival of BLCA patients.** (**A**) A nomogram combined risk score and clinical information. (**B**, **C**) Calibration plot evaluating the predictive accuracy of the nomogram at 3-year (**B**) and 5-year survival (**C**).

In the training set, the 8-IRlncRNA classifier, age and pathological stage were highly associated with OS by univariate and multivariate Cox regression analysis. Except for age, similar results were observed in the validating set (Table 4). Our results showed that 8-IRlncRNA classifier was an independent prognostic factor for OS in BLCA. A nomogram to predict 3- and 5-year overall survival utilizing the 8-IRlncRNA signature, pathological stage and histologic grade was developed (Figure 5A). The concordance index (C-index) of nomogram was 0.71, which increased the predictive power of OS compared with the 8-IRlncRNA classifier (C-index = 0.70). The calibration curves for 3- and 5-year overall survival showed that the predicted probability of OS was approximately equivalent to the actual OS (Figure 5B, 5C).

**Table 4:** Univariate and multivariate Cox regression analysis of the 8-IRlncRNA classifier with OS in the training set and validating set.

**Features**	**Univariate COX**			**Multivariate COX**	
	**HR (95% CI)**	**P**		**HR (95% CI)**	**P**
**Training set**				
Age (>70 vs≤70)	2.459(1.320,4.579)	**0.005**		2.053(1.099,3.836)	**0.024**
Gender (Male vs Female)	0.787(0.523,1.184)	0.250			
Pathological stage (III+IV vs I+II)	2.176(1.386,3.416)	**0.001**		1.988(1.261,3.134)	**0.003**
Histologic grade (High vs Low)	3.844(0.535,27.601)	0.181			
Diagnosis subtype (Papillary vs Non-Papillary)	0.791(0.514,1.216)	0.285			
8-IRlncRNA classifier (High risk vs Low risk)	3.365(2.246,5.043)	**<0.001**		3.236(2.145,4.882)	**<0.001**
**Validating set**					
Age (>70 vs≤70)	1.525(0.813,2.860)	0.189			
Gender (Male vs Female)	0.998(0.582,1.711)	0.993			
Pathological stage (III+IV vs I+II)	2.393(1.247,4.590)	**0.009**		2.015(1.001,4.053)	**0.050**
Histologic grade (High vs Low)	2.008(0.276,14.595)	0.491			
Diagnosis subtype (Papillary vs Non-Papillary)	0.523(0.283,0.964)	**0.038**			
8-IRlncRNA classifier (High risk vs Low risk)	2.182(1.317,3.614)	**0.002**		1.948(1.151,3.296)	**0.013**

Abbreviations: OS, Overall survival; HR, Hazard ratio; CI, Confidence interval.

### Prognostic value of the 8-IRlncRNA classifier for assessing recurrence-free survival

**Figure 6:**
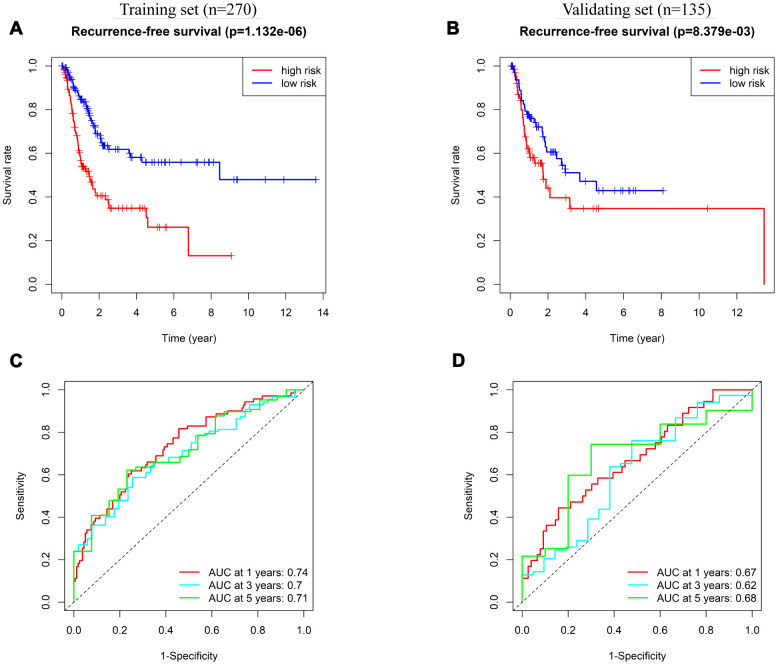
**Prognostic value of 8-IRlncRNA classifier for assessing recurrence-free survival (RFS).** (**A**, **B**) Kaplan-Meier analysis for RFS of BLCA patients based on the risk stratification in the training set (**A**) and validating set (**B**). (**C**, **D**) Receiver operating characteristic (ROC) analysis for RFS prediction including 1-, 3-, 5-year of BLCA patients in the training set (**C**) and validating set (**D**).

We also explored the prognostic value of the 8-IRlncRNA classifier to predict RFS of BLCA. Survival analysis showed that the RFS of patients in the high-risk group was significantly shorter than that in the low-risk group (Figure 6A, 6B). To evaluate the ability of the 8-IRlncRNA classifier to predict RFS of BLCA, we plotted ROC curves, and the AUC values of the classifier for prediction of 1-, 3- and 5-year RFS were 0.74, 0.7, and 0.71, respectively, in the training set (Figure 6C) and 0.67, 0.62, 0.68 respectively in the validating set (Figure 6D). Multivariate Cox regression analysis was conducted to identify prognostic factors for RFS, and outcomes indicated that pathological stage and the 8-IRlncRNA classifier were independent risk factors for RFS in BLCA patients (Supplementary Table 1). Our results demonstrated that this 8-IRlncRNA classifier could be a reliable prognostic predictor of BLCA recurrence.

### Pathway enrichment analysis of the 8-IRlncRNA signature

**Figure 7:**
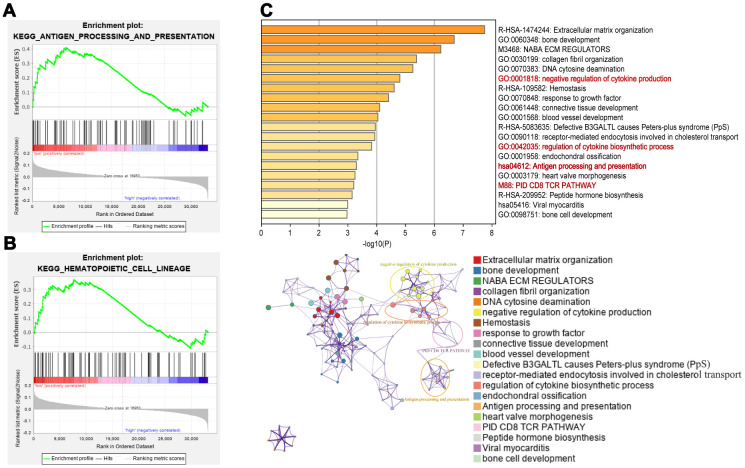
**Pathway enrichment analysis of the 8-IRlncRNA signature.** (**A**, **B**) Immunologic characteristics regulated via the immune-related lncRNA signature, including antigen processing and presentation (**A**) and hematopoietic cell lineage (**B**). (**C**) Pathways associated with the 8-IRlncRNA signature were enriched using genes which expressions were highly correlated with the 8-IRlncRNA signature by Metascape. The upper image showed the histogram of the top 20 enriched pathways associated with the 8-IRlncRNA-based signature. The abscissa was the value of -Log_10_P and longitudinal were different enrichment pathways, sorted by the value of -Log_10_P. The under image showed the network of enriched terms. Each node represented an enriched term and was colored by its cluster ID.

To study the potential molecular mechanism of the 8-IRlncRNA classifier in BLCA, we performed gene set enrichment analysis (GSEA) of the high- and low-risk groups. The results showed that immune-related pathways such as antigen processing and presentation (Figure 7A) and hematopoietic cell lineage (Figure 7B) were inactivated in the high-risk group, suggesting that these pathways were correlated with the disease progression of BLCA. In addition, the top 5% of genes whose expression was correlated with the 8-IRlncRNA signature (p<0.05) were selected for enrichment analysis and some important immune-related pathways, including negative regulation of cytokine production, regulation of cytokine biosynthetic process, antigen processing and presentation, and PID CD8 TCR pathway were enriched (Figure 7C).

### Correlation of the 8-IRlncRNA signature with immune cell infiltration

**Figure 8:**
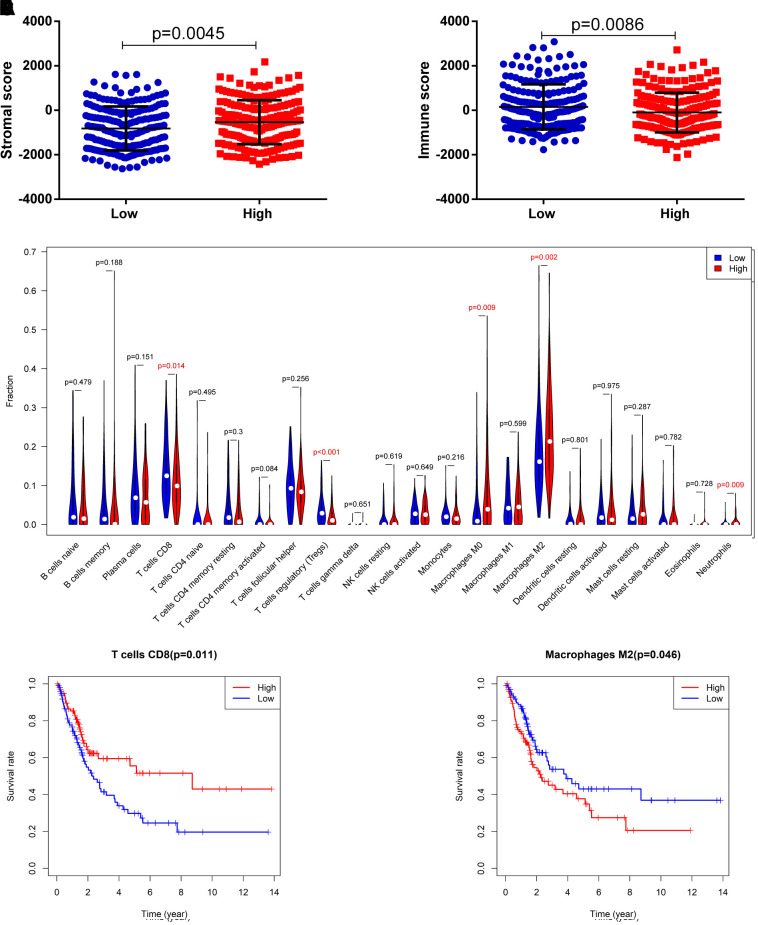
**Correlation of the 8-IRlncRNA signature with immune cell infiltration.** (**A**) The stromal score in the low- and high-risk groups. (**B**) The immune score in the low- and high-risk groups. (**C**) The difference of 22 tumor-infiltrating immune cells among risk groups as defined by the 8-IRlncRNA signature. (**D**, **E**) The survival analysis for the abundance ratios of the T cells CD8 (**D**) and macrophages M2 cells (**E**). The red line indicates a high expressing group of immune cells, and the blue line indicates a low expressing group of immune cells.

It was suggested that this 8-IRlncRNA classifier was related to immune-related pathways. Therefore, we explored the difference in immune cell infiltration between the two groups. Based on the ESTIMATE algorithm, we first calculated the stromal score and immune score of each BLCA sample. Higher stromal scores (-536.2 vs -816.8, p=0.005) and lower immune scores (-99.59 vs 151.2, p=0.009) were observed in the high-risk group compared with the low-risk group (Figure 8A, 8B), indicating different infiltration levels of immune cells in different risk groups. We further analyzed the abundance of 22 tumor-infiltrating immune cells in the two groups. In the high-risk group, the proportions of CD8 T cells (0.1105 vs 0.1371, p=0.014) and regulatory T cells (0.0207 vs 0.0384, p<0.001) were decreased, while the proportions of M0 macrophages (0.0764 vs 0.040, p=0.009), M2 macrophages (0.2323 vs 0.1880, p=0.002) and neutrophils (0.0075 vs 0.0031, p=0.009) were increased compared with those in the low-risk group (Figure 8C). Among these cells, low CD8 T cell infiltration was associated with low OS (Figure 8D, p=0.011) while high macrophage M2 cell infiltration was associated with low OS (Figure 8E, p=0.046). These findings strongly suggest that this IRlncRNA signature is associated with prognosis by interfering with immune cell infiltration in BLCA.

### Potential of the IRlncRNA signature as an indicator of response to immunotherapy

**Figure 9:**
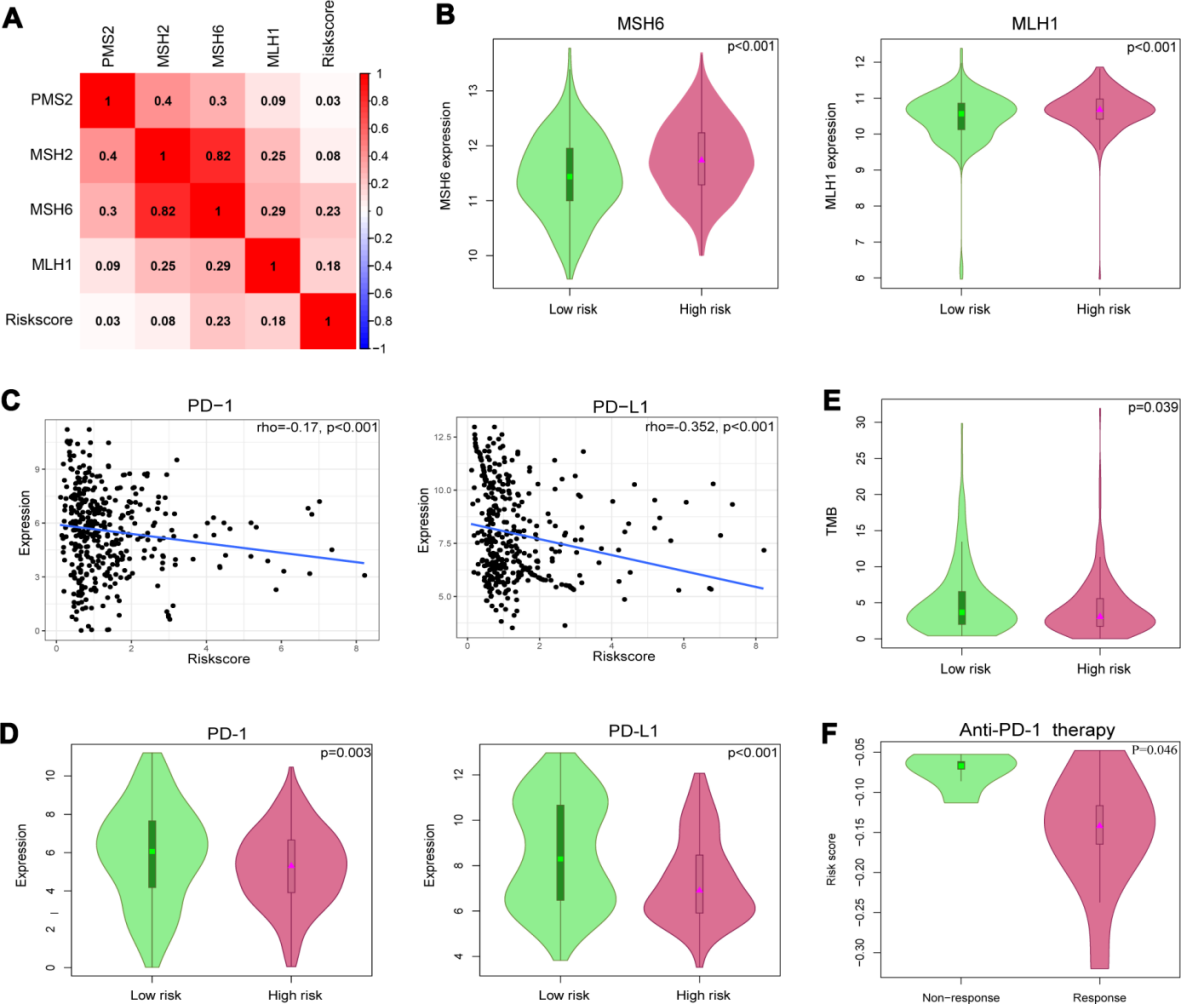
**Identification of 8-IRlncRNA signature for predicting immunotherapeutic response in BLCA.** (**A**) The relationship between the mismatch repair (MMR) genes and risk score defined by the 8-IRlncRNA signature. (**B**) The different expressions of MSH6 and MLH1 among risk groups as defined by the 8-IRlncRNA signature. (**C**) Significant association between our immune-related lncRNA signature and immune checkpoint inhibitors PD-1 and PD-L1. (**D**) The different expressions of PD-1 and PD-L1 among risk groups as defined by the 8-IRlncRNA signature. (**E**) The difference of tumor mutation burden (TMB) among risk groups as defined by the 8-IRlncRNA signature. (**F**) The difference of the risk score in two groups (response vs. non-response to the anti-PD-1 therapy).

Tumor immunotherapy using ICIs has been a promising treatment for advanced urothelial carcinoma [19]. It was confirmed that solid tumors deficient in MMR genes were usually immunogenic and showed extensively infiltrating T cells, making them highly responsive to ICIs [20]. We evaluated the correlation between the IRlncRNA signature and four key MMR genes (MSH6, MLH1, PMS2, MSH2). The results demonstrated that the risk score was significantly positively correlated with the expression of MLH1 (r=0.18, p<0.001) and MSH6 (r=0.23, p<0.001) (Figure 9A). The expression levels of MSH6 and MLH1 were both upregulated in the high-risk group (p<0.001 for MSH6, and p<0.001 for MLH1), indicating that this group benefited less from immunotherapy (Figure 9B). TMB was also a predictive biomarker for immunotherapy, and a high TMB suggested a high response rate to immunotherapy [21]. We acquired single nucleotide variation (SNV) data of 412 BLCA samples from TCGA and then selected the data processed by VarScan software for subsequent analysis. The landscape of mutation data in BLCA is showed in Supplementary Figure 2. We calculated the TMB for BLCA patients and matched the data for patients in our cohort. Patients in the high-risk group had a lower TMB than those in the low-risk group (4.313 vs 5.235, p=0.039), suggesting that these patients may be insensitive to ICIs (Figure 9E). Then, the association between the IRlncRNA signature and the expression levels of immune checkpoint genes (PD-1 and PD-L1) was investigated. As shown in Figure 9C, the risk score was significantly negatively correlated with the expression of PD-1 (r=0.17, P<0.001) and PD-L1 (r=0.352, p<0.001). The expression levels of PD-1 and PD-L1 were both downregulated in the high-risk group (p=0.003 for PD-1, and p<0.001 for PD-L1) (Figure 9D). These observed associations between our IRlncRNA signature and immunotherapy-related biomarkers indicated that BLCA patients in the high-risk group may be insensitive to ICIs. Therefore, the predictive value of the IRlncRNA signature was tested in the clear cell renal cell carcinoma (ccRCC) immunotherapy dataset [22]. The results demonstrated that patients in the nonresponse group had a higher risk score as defined by the 8-IRlncRNA signature (Figure 9F, p=0.046).

## DISCUSSION

BLCA is a complex disease with high morbidity and mortality rates if not treated properly [19]. Therefore, the early diagnosis and prognostic prediction of patients with BLCA are important. Diverse models to predict the outcome of BLCA, including miRNA-based signatures [23, 24], clinical character-based nomograms [25], and lncRNA-based models [26], have been reported. Accumulating evidence suggests that lncRNAs play a key role in the process of tumorigenesis, development and metastasis and show potential as novel biomarkers [11, 27]. LncRNA-based signatures have been validated to predict the survival or recurrence of BLCA [28–30]. However, the potential role of immune-related lncRNA signatures as an effective therapeutic strategy in BLCA is unknown.

Here, we identified an 8-IRlncRNA classifier and explored its prognostic value to predict OS and RFS of BLCA, as well as its role in evaluating the response of BLCA patients to ICIs therapy. Among these 8 IRlncRNAs, PTPRD-AS1 has been identified as a reliable signature predicting survival of patients with bladder urothelial carcinoma [31]. LINC01013 enhances the invasion of anaplastic large-cell lymphoma by activating of the epithelial-to-mesenchymal transition [32]. LncRNA MRPL23-AS1 promoted adenoid cystic carcinoma lung metastasis by forming an RNA-protein complex with enhancer of zeste homolog 2 (EZH2) and increasing the binding of EZH2 and H3K27me3 on the E-cadherin promoter region [33]. These studies supported our 8-IRlncRNA classifier as a potentially measurable proxy for the prognosis of cancer patients. Each patient was assigned a score by our classifier and then classified into two categories based on the median risk score. Patients in the high-risk group had shorter survival times than those in the low-risk group. Moreover, our 8-IRlncRNA classifier presented a strong ability to predict OS of BLCA; the AUC values to predict 1-, 3- and 5-year overall survival were 0.72, 0.76, and 0.76, respectively, in the training set and 0.74, 0.68, 0.75 respectively in the validating set. Additionally, we explored the efficiency of the 8-IRlncRNA classifier in predicting RFS of BLCA. The AUC values of the classifier to predict 1-, 3- and 5-year RFS were 0.74, 0.7, and 0.71, respectively, in the training set and 0.67, 0.62 and 0.68, respectively, in the validating set, indicating that our classifier also had potential application value in predicting bladder cancer recurrence.

To explore the biological function of the 8-IRlncRNA signature, we performed GSEA, and the results showed that our IRlncRNA signature may be involved in antigen processing and presentation and hematopoietic cell lineage. In addition, some immune -related signaling pathways involved in the negative regulation of cytokine production, the regulation of cytokine biosynthetic processes, antigen processing and presentation, and the PID CD8 TCR pathway were enriched by using genes that were highly correlated with our IRlncRNA signature. It has been reported that the tumor immune microenvironment has significant value in the prognostic study of MIBC [34]. In our study, patients in the high-risk group had low CD8 T cells and high macrophage M2 infiltration in the microenvironment, indicating that our 8-IRlncRNA classifier may interfere with immune cell infiltration in BLCA. The exact mechanisms of these IRlncRNAs remain largely unknown, and more research is required to investigate their roles in BLCA.

Fifty percent of MIBC patients relapse and often have distant metastases after radical surgery. Cisplatin-based combination chemotherapy is the standard first-line treatment for metastatic patients with good renal function [35]. However, increasing resistance limits the chemotherapy efficacy of these patients. Recently, immunotherapy has afforded a promising new treatment option for metastatic BLCA [36], but only 20% -30% of patients with advanced bladder cancer were responsive to immunotherapy. Therefore, novel predictive biomarkers for immunotherapy need to be identified. High TMB and neoantigen load were associated with a high response to ICIs. In our study, the correlation analysis demonstrated that the 8-IRlncRNA signature was positively related to the MMR genes MSH6 and MLH1, and patients in the high-risk group had a low TMB, revealing that these patients may respond poorly to ICIs. In addition, the 8-IRlncRNA signature was significantly negatively correlated with the expression of immune checkpoint genes (PD-1 and PD-L1). Moreover, low CD8 T cells and high macrophage M2 infiltration in the high-risk group were previously shown to be associated with poor response in immunotherapy [37]. These results suggested that the 8-IRlncRNA signature could serve as a potential biomarker for measurement of the response to ICIs treatment.

In conclusion, we identified 8 IRlncRNAs associated with OS in BLCA and constructed an 8-IRlncRNA classifier for prognostic prediction. This 8-IRlncRNA classifier also demonstrated considerable predictive accuracy for predicting RFS. In addition, the 8-IRlncRNA signature is correlated with immunotherapy-related biomarkers, suggesting its application value in predicting the efficacy of immunotherapy. This study is the first report that an IRlncRNA signature could predict prognosis and immunotherapeutic response in human bladder cancer. Nevertheless, large-scale, multicenter and prospective studies are necessary to confirm our results before the 8-IRlncRNA signature can be applied in the clinic.

## MATERIALS AND METHODS

### Data acquisition

Gene expression quantification data (FPKM and counts format) and SNV data for BLCA were downloaded from TCGA (https://portal.gdc.cancer.gov/). Then 19 normal samples and 411 BLCA samples were obtained. The matrix of RNA expression was extracted separately by annotations using the Gencode (GENCODE v 26) GTF file and normalized. Genes whose expression was “zero” in 90% of BLCA patients were removed. Clinical data were downloaded from the UCSC Xena website (https://xena.ucsc.edu/). To analyze the correlation of lncRNA expression signatures with the prognosis of bladder cancer patients, we filtered out samples without survival information. Then, we selected a total of 405 patients, and these patients were randomly divided into training (n=270) and validating sets (n=135) randomly at a 2:1 ratio for further analysis. Significant lncRNA-pathway pairs across 33 cancer types with each lncRNA having an activity in immune pathways (lncRES) score> 0.995 and a false discovery rate (FDR) < 0.05 were downloaded from Immlnc (http://biobigdata.hrbmu.edu.cn/ImmLnc/index.jsp) [38]. The list of immune-related lncRNAs in BLCA was extracted separately. Stromal scores and immune scores of BLCA were calculated by applying the ESTIMATE algorithm and downloaded from the website (https://bioinformatics.mdanderson.org/estimate/index.html) [39]. TMB was defined as the total number of somatic mutations per million bases and analyzed by the R package ‘maftools’. Because the ICIs treatment dataset was not available in BLCA samples, a dataset of ccRCC with available ICIs treatment data and transcriptomic profiles was obtained from the study of Miao et al [22]. Fifteen patients treated with PD-1 blockade therapy remained after excluding patients without expression of the 8 IRlncRNAs.

### Analysis of differentially expressed lncRNAs

We obtained DElncRNAs between normal and tumor tissues, where P value <0.05 and |log2-fold change (FC)|> 1 were used as the cutoffs by using the R package ‘edgeR’ [40]. Then, we filtered DEIRlncRNAs by matching the list of immune-related lncRNA in BLCA. The R package ‘heatmap’ was used to display the eight selected IRlncRNAs.

### Data processing and risk score calculation

First, the DEIRlncRNAs were subjected to univariable Cox regression analysis to select IRlncRNAs that were associated with the OS of BLCA patients. Final 42 IRlncRNAs with a p value<0.05 were found. Second, we conducted LASSO regression analysis to identify more meaningful prognostic variables. Finally, we used these IRlncRNAs in multivariable Cox regression to obtain the coefficients. Eight IRlncRNAs significantly correlated with OS were identified to build the prediction model weighted by their coefficients. A risk-score formula for OS was constructed, and each patient was assigned a risk score by this risk-score formula that was a linear combination of the expression levels of significant IRlncRNAs weighted by their respective Cox regression coefficients. The patients were divided into a low-risk group and a high-risk group based on the median risk score.

### Identifying survival-related immune cells

Cell-type Identification By Estimating Relative Subsets Of RNA Transcripts (CIBERSOFT) is an analytical tool utilizing deconvolution algorithm that can infer 22 human immune cell types and quantify the relative ratio of each cell type (http://cibersort.stanford.edu). To enhance the robustness of the results, CIBERSORT produces an empirical P value for the deconvolution of each sample based on Monte Carlo sampling [41]. RNA-Seq (FPKM format) data of BLCA was analyzed by R software to obtain the abundance ratio matrix of 22 immune cells in each sample. Samples with p > 0.05 were filtered out to increase the accuracy of the estimated results.

### Pathway enrichment analysis

To explore the potential functions of the eight-IRlncRNA signature, we conducted GSEA to assess whether a predefined set of genes showed statistically significant, concordant differences according to the risk group by GSEA software (downloaded from http://software.broadinstitute.org/gsea/index.jsp) [42]. The gene database of “c2.cp.kegg.v6.2.symbols.gmt” from the molecular signature database was analyzed. Pathway enrichment analysis was conducted using the online database “Metascape” (http://metascape.org/) [43]. The significance threshold of FDR for enriched biological processes and pathways was set at 0.05.

### Statistical analysis

The chi-square test or Fisher's exact test was conducted to measure the difference between the training and validating sets and the relationship between clinical data and risk score. Spearman's correlation coefficients were computed to investigate the potential relationship between two groups. Both univariable and multivariable Cox regression analyses were performed using the R package ‘survival’. The Kaplan-Meier survival curve with log-rank test was drawn to demonstrate the relationship between IRlncRNAs and OS or RFS by the R package ‘survival’. The Wilcoxon rank-sum test is a nonparametric statistical test mainly utilized for comparing two groups. The ROC curve was generated to measure the accuracy of survival prediction by the R package ‘survivalROC’. All statistical tests were two-sided, and p < 0.05 was considered statistically significant. All analyses were performed in SPSS version 25.0 (SPSS Inc., Chicago, IL, USA) or R version 3.5.2 (http://www.r-project.org/).

## Supplementary Material

Supplementary Figures

Supplementary Table 1
