# Supplementary material for: Identification of immune-related LncRNA for predicting prognosis and immunotherapeutic response in bladder cancer
Source: Aging (Albany NY). 2020 Nov 18;12(22):23306–25. doi: 10.18632/aging.104115 (PMC7746369; doi:10.18632/aging.104115)
Supplement: Supplementary Figures [file aging-12-104115-s001..pdf]

SUPPLEMENTARY FIGURES

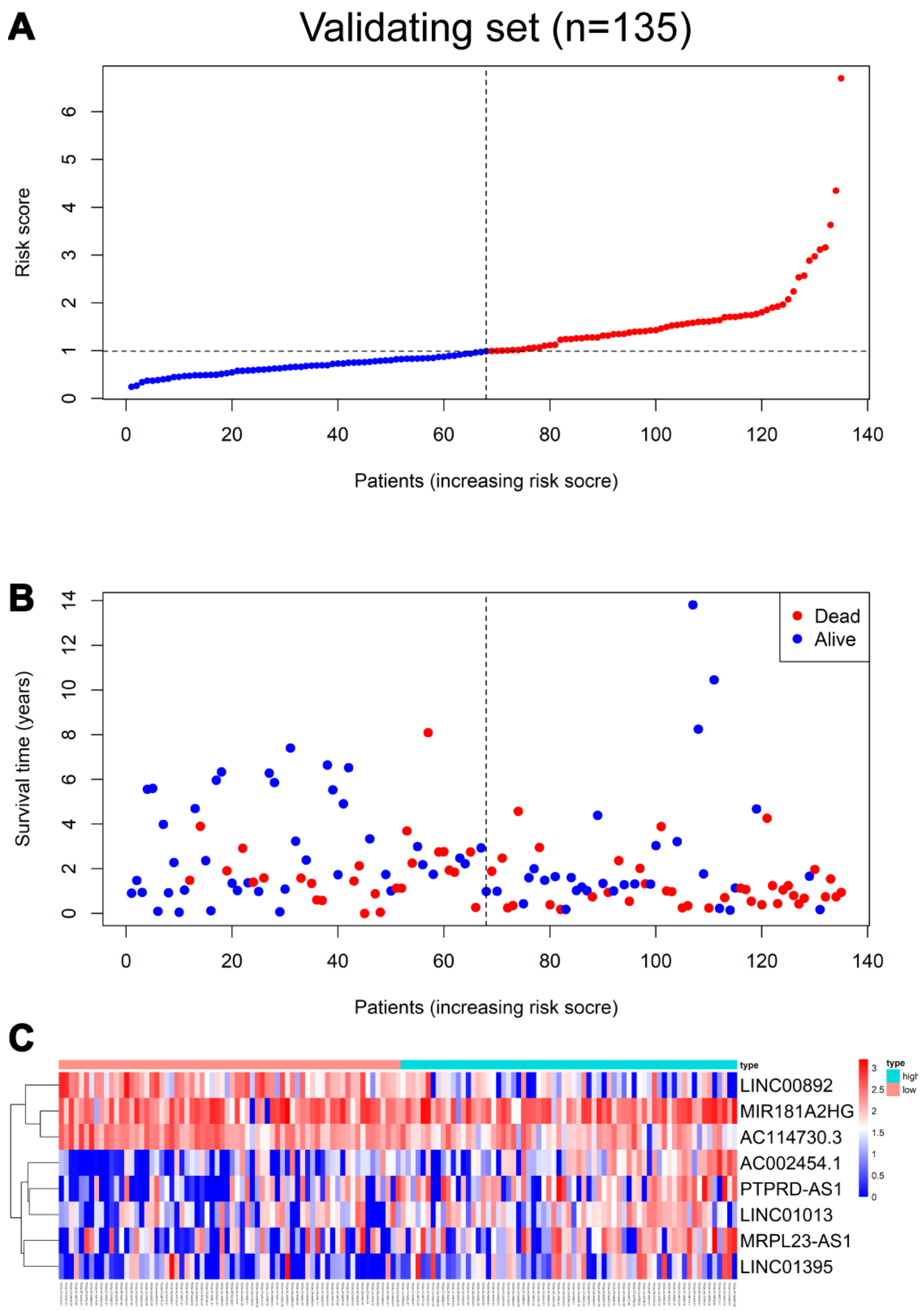

Supplementary Figure 1. Construction of the 8-IRlncRNA classifier for predicting prognosis in BLCA (**A**) Patients with BLCA were sorted by increasing risk score in the validating set. (**B**) Living status of BLCA patients in the validating set. (**C**) Heatmap of eight IRlncRNAs expression profiles of different risk groups in the validating set.

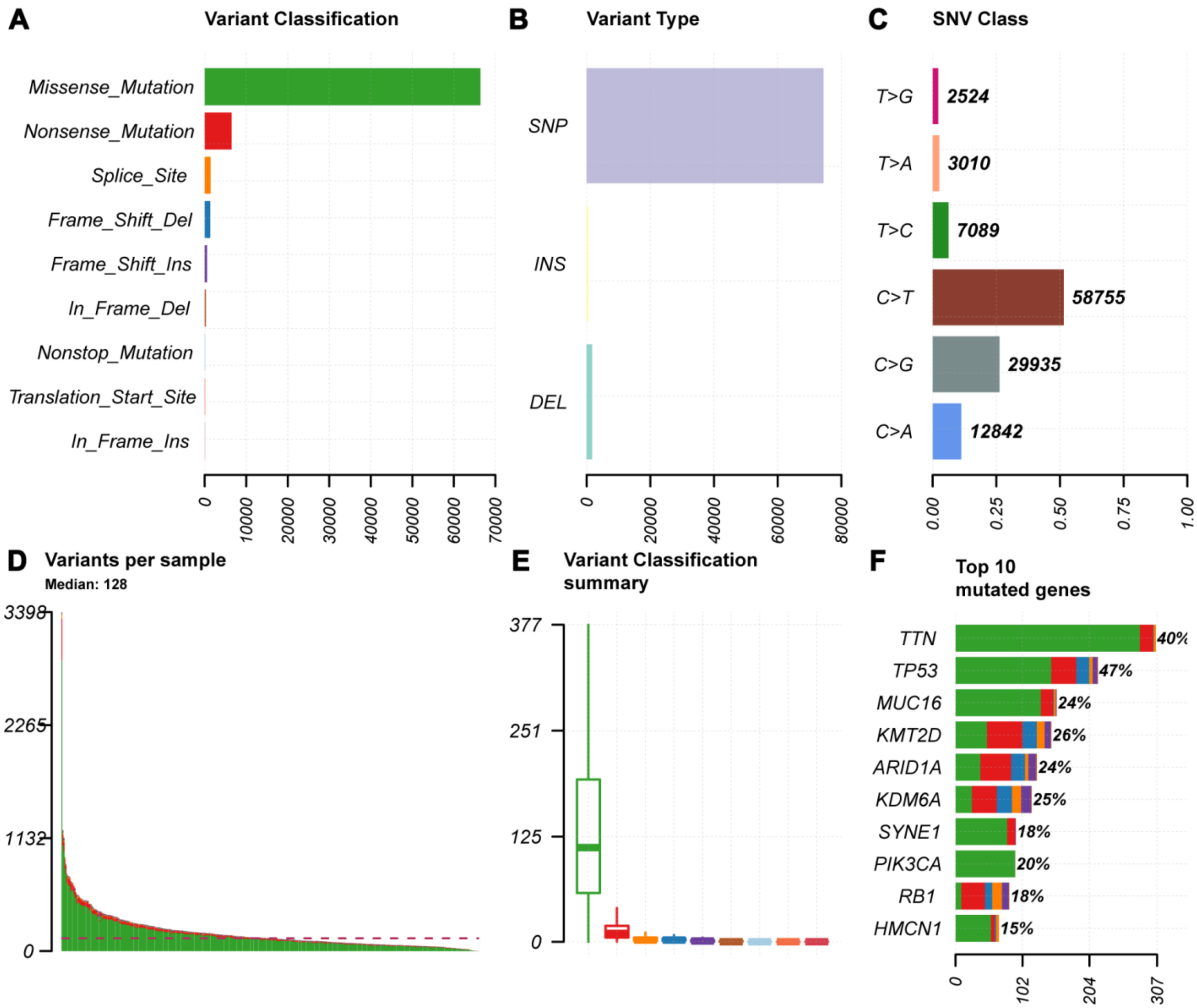

Supplementary Figure 2. Summary of mutation profiling in BLCA samples. (**A**–**C**) Statistical calculation of mutation types based on different categories, where missense mutation occupies the most component, SNP exhibited more frequent than the deletion or insertion, and C > T was the most common class of single nucleotide variation (SNV). (**D**, **E**) Summary of variant number and classification in specific samples. (**F**) The top 10 mutated genes in BLCA.
