# Supplementary material for: Identification of immune-related LncRNA for predicting prognosis and immunotherapeutic response in bladder cancer
Source: Aging (Albany NY). 2020 Nov 18;12(22):23306–25. doi: 10.18632/aging.104115 (PMC7746369; doi:10.18632/aging.104115)
Supplement: Supplementary Table 1 [file aging-12-104115-s002..pdf]

SUPPLEMENTARY TABLE

Supplementary Table 1. Univariate and multivariate Cox regression analysis of the 8-IRlncRNA classifier with RFS in TCGA-BLCA database.

Features	Univariate COX		Multivariate COX	
	HR (95% CI)	P	HR (95% CI)	P
Age (>70 vs≤70)	1.068(0.787,1.449)	0.672		
Gender (Male vs Female)	0.914(0.652,1.282)	0.603		
Pathological stage (III+IV vs I+II)	2.177(1.512,3.136)	**<0.001**	1.968(1.357,2.854)	**<0.001**
Histologic grade (High vs Low)	3.526(0.873,14.243)	0.077		
Diagnosis subtype (Papillary vs Non-Papillary)	0.635(0.442,0.913)	**0.014**		
8-IRlncRNA classifier (High risk vs Low risk)	2.154(1.582,2.932)	**<0.001**	2.052(1.497,2.813)	**<0.001**

Abbreviations: RFS, Recurrence free survival; TCGA, The Cancer Genome Atlas; BLCA, Bladder cancer; HR, Hazard ratio; CI, Confidence interval.
